# Flexible quantitative bias analysis for unmeasured confounding in subject-level indirect treatment comparisons with proportional hazards violation

**DOI:** 10.1186/s12874-025-02551-z

**Published:** 2025-05-10

**Authors:** Steven Soutar, Amy Macdougall, Jamie Wallis, Joseph E. O’Reilly, Lewis Carpenter

**Affiliations:** Arcturis Data, Building One, Oxford Technology Park, Technology Drive, Oxford, OX5 1GN UK

**Keywords:** Indirect treatment comparisons, Non-proportional hazards, Unmeasured confounding, Quantitative bias analysis, Bayesian data augmentation, Multiple imputation, Restricted mean survival

## Abstract

**Background:**

Indirect treatment comparisons can provide evidence of relative efficacy for novel therapies when implementation of a randomised controlled trial is infeasible. However, such comparisons are vulnerable to unmeasured confounding bias due to incomplete data collection and non-random treatment assignment. Quantitative bias analysis (QBA) is a framework used to assess the sensitivity of a study’s conclusions to unmeasured confounding. As indirect comparisons between therapies with differing treatment modalities may result in violation of the proportional hazards (PH) assumption, QBA methods that are applicable in this context are required. However, few QBA methods are valid under PH violation.

**Methods:**

We proposed a simulation-based QBA framework which quantifies the sensitivity of the difference in restricted mean survival time (dRMST) to unmeasured confounding, and is therefore valid under violation of the PH assumption. The proposed framework utilises Bayesian data augmentation for the multiple imputation of an unmeasured confounder with user-specified characteristics. Adjustment of dRMST is then implemented in a weighted analysis using the imputed values. The accuracy and precision of our proposed imputation-based adjustment method was assessed through a simulation study. Confounded data was simulated using a common non-PH data generating process, and imputation-based effect estimates were compared against estimates obtained following adjustment for all confounders. Implementation of the proposed QBA framework was also illustrated using a data from an external control arm study demonstrating clear PH violation.

**Results:**

Imputation-based adjustment using Bayesian data augmentation was observed to estimate the true adjusted dRMST with minimal bias. Moreover, the bias was comparable to that observed under adjustment when all confounders were measured. Application of the proposed QBA framework to an indirect treatment comparison study enabled identification of the characteristics of an unmeasured confounder that would be required to nullify the study’s conclusions.

**Conclusions:**

Imputation-based adjustment can accurately recover the true adjusted dRMST in the presence of unmeasured confounding with known exposure and outcome associations. Therefore, the proposed QBA framework can correctly determine the characteristics required by an unmeasured confounder to invalidate a study’s conclusions. Consequently, this framework enables the construction of sensitivity analyses to investigate the robustness of relative efficacy evidence derived from indirect treatment comparisons which exhibit PH violation.

**Supplementary Information:**

The online version contains supplementary material available at 10.1186/s12874-025-02551-z.

## Background

Demonstration of the efficacy of a novel treatment relative to an established therapy is a fundamental requirement of both the regulatory and reimbursement assessment process. The randomised controlled trial (RCT) is considered the gold standard for generating such evidence [1], but in certain situations it may not be possible to perform an RCT for ethical or logistical reasons [2]. In these scenarios the implementation of a single-arm trial [2], in which patients only receive the novel intervention, allows for assessment of treatment response and safety, but with no demonstration of comparative efficacy. Relative efficacy can still be demonstrated using a single-arm study through the increasingly common method of indirect treatment comparison (ITC) [3], which involves comparing outcomes from the clinical study to an external data source, such as a historical trial or an electronic health record (EHR) database [3–6].

ITC methods are applicable both when individual-level patient data (IPD) from the external source is unavailable [7, 8] and when IPD is accessible. In the latter case, an external control arm (ECA) can be constructed, allowing combined analysis of outcomes and covariates from the clinical study and the external source [9–11]. In addition to contextualising results from single-arm trials, another common application of ECA analysis is estimation of the efficacy of a novel therapy relative to a comparator not included in the control arm of a phase III RCT. This is done by comparing patient-level data from the intervention arm of an RCT to an ECA consisting of a population treated with a different control therapy than the one used in the RCT.

Despite the ability of ITCs to generate evidence in the absence of an RCT, their application requires careful consideration [12]. A particular concern with any ITC is the presence of confounding bias, which occurs when patient characteristics (confounders) have an association with both treatment assignment and the outcome of interest. Random treatment assignment, as used in an RCT, ensures that measured and unmeasured confounders are balanced between arms, protecting against bias when outcomes are compared between the study arms [13]. The absence of randomisation in an ITC means that confounding bias is likely when outcomes are naively compared [14]. Identification and measurement of confounders enables statistical methods to account for their influence [14–18]. However, even after adjustment for measured confounders, the presence of unmeasured confounding remains a concern in non-randomised studies, particularly when using EHR data, where the measurement of confounders depends on relevance to clinical decision-making.

The application of quantitative bias analysis (QBA) has been recommended to assess the potential impact of unmeasured confounding on a study’s conclusions [19–21]. QBA is often conducted as a tipping point analysis, identifying exposure and outcome associations of a hypothetical unmeasured confounder that would render the focal effect estimate non-significant. If the identified associations are considered implausible then the study is likely to be robust to unmeasured confounding. Several QBA methods have been proposed [22–25] and can be grouped into two categories: bias-formula methods, including the popular E-value approach [26], which directly compute confounder-adjusted effect estimates [26–28], and simulation-based approaches [29–32], which treat unmeasured confounding as a missing data problem solved through imputation of unmeasured confounders, which are then applied in a adjusted analysis [31–33]. Bias-formula methods have the advantage of being relatively easy to implement and interpret, but are limited to specific scenarios of unmeasured confounding [34]. In contrast, simulation-based methods offer greater flexibility by allowing users to construct specific confounding scenarios of interest, but potentially require advanced statistical expertise to ensure proper implementation and interpretation of results.

To accurately assess the robustness of conclusions obtained from an ITC using QBA, the chosen framework must be compatible with modelling assumptions used to perform the outcome comparison which generated the focal treatment effect estimate. Many key outcomes for assessments of a novel therapy are time-to-event (TTE) measures, such as time to disease progression, as they often capture the duration of a therapy’s effectiveness. When ITC derived evidence is generated for a TTE outcome, the proportional hazards (PH) assumption is commonly relied upon, but is often violated in practice due to comparisons between therapies with unique methods of action [35–40]. The dRMST, defined as the difference in mean survival time between treatment groups up to a user-specified time horizon, has been recommended as a valid effect measure in the presence of PH violation [41, 42]. Both parametric and non-parametric estimators for dRMST have been proposed [43, 44], with re-weighting schemes available for adjustment of dRMST for measured confounders [45, 46]. Valid assessment of ITC derived TTE evidence requires effect measures and sensitivity analyses which are applicable under PH violation. Despite this, The majority of QBA methods for TTE outcomes, with the exception of the dRMST based methods proposed by Lin et al. and Lee et al. [47, 48], require the PH assumption to be satisfied [49–51].

With the frequent occurrence of PH violation, particularly in oncology immunotherapy studies [39, 52]—which are a growing focus of HTA assessments—a flexible QBA method valid under PH violation would be invaluable for assessing the robustness of ITC analyses. In this work we propose a method consisting of a simulation-based QBA framework to assess the sensitivity of dRMST to potential unmeasured confounding. In contrast to earlier simulation-based QBA frameworks that rely on likelihood-based imputation methods [29, 30], our proposed framework employs a Bayesian imputation approach. This allows for greater flexibility in handling processes that generate non-proportional hazards (non-PH) data, accommodating different distributional forms of unmeasured confounding, and can be implemented and extended using widely available software packages.

## Methods

In this section we first present the specifics of the proposed QBA framework, before describing two analyses utilising the framework, specifically, an assessment of the validity of adjusted treatment effect estimates in a simulation study, and an application using a dataset from a published ITC analysis demonstrating clear violation of the PH assumption.

### Outline

The proposed QBA framework takes the form of a tipping point analysis where estimation of the dRMST, adjusted for potential unmeasured confounding, is performed as a two-step process. In step one, unmeasured confounding is treated as a missing data problem, and multiple imputation (MI) of the unmeasured confounder is performed using user-specified outcome and exposure associations. This MI process utilises Bayesian data augmentation as its methodological foundation. In step two, multiple adjusted analyses are performed using the imputed values and adjusted effect estimates are subsequently pooled and consolidated into a single estimate and associated confidence interval. The two-step process is iterated across a range of user-specified associations to identify associations that are sufficient to nullify the study’s conclusions.

### Proposed QBA framework

#### Step 1: multiple imputation of unmeasured confounders

We now provide a general description of the MI method used to obtain values of an unmeasured confounder. We begin by establishing some notation and assumptions. First, let $i = 1,\dots ,N$ index patients in the study cohort. Now let ${t}_{i}$ denote the (possibly censored) outcome for patient $i$, and ${\updelta }_{i}\in \{\text{0,1}\}$ an event indicator where ${\updelta }_{i}=1$ indicates that patient $i$ experienced the event, and ${\delta }_{i}=0$ otherwise. Now let ${z}_{i}\in \{\text{0,1}\}$ denote a treatment indicator where ${z}_{i}=1$ indicates membership of the intervention arm, and ${z}_{i}=0$ otherwise. Finally, let ${x}_{i}$ denote a vector of measured confounders and ${u}_{i}\in \{\text{0,1}\}$ an (unmeasured) confounder. Although a binary form is assumed for ${u}_{i}$, a continuous variable representing the combined effect of multiple unmeasured confounders is also plausible [28, 53]. For notational convenience we define ${\varvec{x}}=\left\{{x}_{1},\dots ,{x}_{N}\right\}, {\varvec{u}}=\{{u}_{1},\dots ,{u}_{N}\}, {\varvec{z}}=\{{z}_{1},\dots ,{z}_{N}\}$, ${\varvec{\updelta}}=\{{\updelta }_{1},\dots ,{\updelta }_{N}\}$, and ${\varvec{t}}=\{{t}_{1},\dots ,{t}_{N}\}$. The following outcome model $f$ and propensity model $g$ are assumed:

1 $$f\left({t}_{i} | {z}_{i}, {x}_{i}, {u}_{i},{\delta }_{i},{\varvec{\theta}},{{\varvec{\beta}}}_{x},{{\varvec{\beta}}}_{u}\right),$$

2 $$g\left({z}_{i} |{ {x}_{i}, u}_{i}, {\boldsymbol{\alpha }}_{x},{\boldsymbol{\alpha }}_{u}\right).$$

In Eq. (1) the effect of treatment in both arms is captured by the parameter vector ${\varvec{\theta}}$**,** with the effect of measured and unmeasured confounders on survival denoted by the parameter vectors ${{\varvec{\beta}}}_{x}$ and ${{\varvec{\beta}}}_{u}$, respectively. In Eq. (2) the effect of measured and unmeasured confounders on treatment assignment are denoted by the parameter vectors ${\boldsymbol{\alpha }}_{x}$ and ${\boldsymbol{\alpha }}_{u}$, respectively. In contrast to ${\varvec{\theta}}$, ${{\varvec{\beta}}}_{x}$, and ${\boldsymbol{\alpha }}_{x}$, which are estimated, ${{\varvec{\beta}}}_{u}$ and ${\boldsymbol{\alpha }}_{u}$ are known parameters which are varied by the user to assess the sensitivity of the study’s conclusions under different unmeasured confounding scenarios.

In principle, the proposed framework imposes no restrictions on the specification of $f$, which may represent any parametric model, whether it adheres to or deviates from the proportional hazards assumption. In the simplest case of a proportional hazards model with exponential survival, ***θ*** would encompass the baseline hazard and the log hazard ratio, conditioned on any confounding covariates. When violations of the proportional hazards assumption occur, a wide range of options for $f$ are available. These include models incorporating interactions between treatment and time to capture a waning or delayed treatment effect, or the use of a cure model to accommodate long-term survival. If further flexibility is required, modelling the baseline hazard or a time-dependent treatment effect with spline-based approaches would also be viable.

Bayesian data augmentation is used to construct a probabilistic MI scheme for ${u}_{i}$. Data augmentation refers to a class of statistical methods for tractable inference in the presence of missing data or latent variables [54], which have been successfully applied in both frequentist and Bayesian settings [55, 56]. Bayesian data augmentation allows for the specification of the joint posterior over both unknown parameters and the unobserved data, allowing for the inclusion of unobserved data into an MCMC sampling scheme where the posterior samples of parameters representing unobserved data are treated as imputations [56]. In our imputation scheme the unobservable data is the unmeasured confounder ${u}_{i}$.

The joint posterior is defined by first specifying the joint distribution of ${\varvec{t}}$ and ${\varvec{u}}$**,** and then placing priors on ${\varvec{\theta}}$ and ${u}_{i}$ and any hyperparameters. Therefore, we first specify a Bernoulli prior $p\left({u}_{i} \right| \varphi )$, where the hyperparameter $\varphi$ denotes the prevalence of ${u}_{i} = 1$ in the study population. The joint distribution of ${\varvec{t}}$ and ***u***, denoted by $l$, then factorises as:

3 $$l\left({\varvec{t}}, {\varvec{u}}|{\varvec{z}},{\varvec{x}},{\varvec{\delta}}, {\varvec{\theta}},{{\varvec{\beta}}}_{x}, {\boldsymbol{\alpha }}_{x}, {{\varvec{\beta}}}_{u},{\boldsymbol{\alpha }}_{u}\right)= f\left({\varvec{t}}|{\varvec{z}},{\varvec{x}}.{\varvec{u}},{\varvec{\delta}},{\varvec{\theta}},{{\varvec{\beta}}}_{x},{{\varvec{\beta}}}_{u}\right)g\left({\varvec{z}}|{\varvec{x}},{\varvec{u}}, {\boldsymbol{\alpha }}_{x}, {\boldsymbol{\alpha }}_{u}\right)p\left({\varvec{u}}|\varphi \right).$$

The prior specification is then completed by specifying priors $p({\varvec{\theta}})$, $p({{\varvec{\beta}}}_{x})$, $p({\boldsymbol{\alpha }}_{x})$ and $p(\varphi )$. Eq. (3) is then combined with the prior specification using Bayes theorem, to give the joint posterior $\uppi$, which is proportional to:

$$\pi ({\varvec{\theta}},{{\varvec{\beta}}}_{x}, {\boldsymbol{\alpha }}_{x},{\varvec{u}}|{\varvec{t}},{\varvec{x}}\boldsymbol{ },{\varvec{z}},\boldsymbol{ }{\varvec{\delta}},{{\varvec{\beta}}}_{u}, {\boldsymbol{\alpha }}_{u})\propto f({\varvec{t}}|{\varvec{x}},{\varvec{z}},\boldsymbol{ }{\varvec{u}},\boldsymbol{ }{\varvec{\delta}},{\varvec{\theta}},{{\varvec{\beta}}}_{x},{{\varvec{\beta}}}_{u})g({\varvec{z}}|{\varvec{x}},{\varvec{u}},{{\varvec{a}}}_{x},{\boldsymbol{\alpha }}_{u})p({\varvec{u}}|\varphi )p({\varvec{\theta}})p({{\varvec{\beta}}}_{x})p({\boldsymbol{\alpha }}_{x})p(\varphi ).$$

Using Markov chain Monte Carlo (MCMC) methods a sample can be drawn from the joint posterior $\pi$ [57]. When examining the MCMC output our interest is in the marginal posterior sample for ${u}_{i}$, as these represent imputed values for ${u}_{i}$, given the user-specified association parameters.

#### Step 2: imputation-based adjustment for the dRMST

Having extracted a posterior sample, $K$ of the samples for ${u}_{i}$ are retained as imputations and the adjusted dRMST is estimated using numerical integration of weighted Kaplan-Meier (KM) curves [45], where the per-patient weights are calculated conditional on the imputed values of the unmeasured confounder. For each set of $K$ imputations ${{\varvec{u}}}^{k}=\{{u}_{1}^{k},...,{u}_{N}^{k}\}$ a set of weights ${{\varvec{w}}}^{k}=\{{w}_{1}^{k},...,{w}_{N}^{k}\}$ are computed using:

4 $${w}_{i}^{k}=\frac{1}{{z}_{i}g(z = 1|{u}_{i}^{k}, {x}_{i}, {\boldsymbol{ }\boldsymbol{\alpha }}_{x}^{k}, {\boldsymbol{ }\boldsymbol{\alpha }}_{u}) + {(1 - z}_{i})(1 - g(z = 1|{u}_{i}^{k},{\boldsymbol{ }{x}_{i}, {\boldsymbol{ }\boldsymbol{\alpha }}_{x}^{k},\boldsymbol{ }\boldsymbol{ }\boldsymbol{\alpha }}_{u}))}.$$

Note that in Eq. (4) the propensity score for patient $i$ computed using the ${k}^{\text{th}}$ imputation for ${u}_{i}$ is denoted by $g(z = 1|{u}_{i}^{k},{{\boldsymbol{\alpha }}_{x}^{k},\boldsymbol{ }\boldsymbol{\alpha }}_{u})$, where ${\boldsymbol{\alpha }}_{x}^{k}$ denotes the $k^{\text{th}}$ posterior sample for ${\boldsymbol{\alpha }}_{x}$. Using the method of Cole and Hernan [45], the weights ${{\varvec{w}}}^{k}$ are applied to estimate the adjusted dRMST, with variance estimates computed using the method proposed by Conner et al. [46] (see supplementary material for further details).

Weighting using imputations of $u$, yields $K$ point estimates of the adjusted dRMST, denoted by ${\varvec{\gamma}}\boldsymbol{ }=\{{\gamma }_{1},..,{\gamma }_{K}\}$, and associated variance estimates, denoted by ${\varvec{\eta}}=\{{\eta }_{1},...,{\eta }_{K}\}$. To compute pooled adjusted estimates $\widehat{\gamma }$ and $\widehat{\eta }$, and to appropriately propagate uncertainty in the imputations for $u$, we apply Rubin’s rules [58] given by Eqs. (5) and (6) below:

5 $$\widehat{\gamma }= \sum_{k=1}^{K}{\gamma }_{k},$$

6 $$\widehat{\eta }=\frac{1}{K}\sum_{k=1}^{K}{\eta }_{k}+\left(1+ \frac{1}{K}\right)\left(\frac{1}{K-1}\right)\sum_{k=1}^{K}{({\gamma }_{k}-\widehat{\gamma })}^{2}.$$

A pooled confidence interval (CI) is computed using a Student’s t-distribution with the appropriate degrees of freedom.

In principle, our framework accommodates multiple methods for adjusting for ${u}_{i}$ using its imputed values. Here, we have illustrated an approach using inverse probability of treatment (IPT) weighting. However, alternative techniques, such as propensity score matching (PSM), are equally applicable. If PSM is used, a matching process using estimated propensity scores conditioned on the imputed values of ${u}_{i}$ would be performed. The adjusted dRMST would be computed through numerical integration of Kaplan-Meier curves estimated using the matched data, and the variance computed using the estimator proposed by Lin et al. [47].

**Fig. 1:**
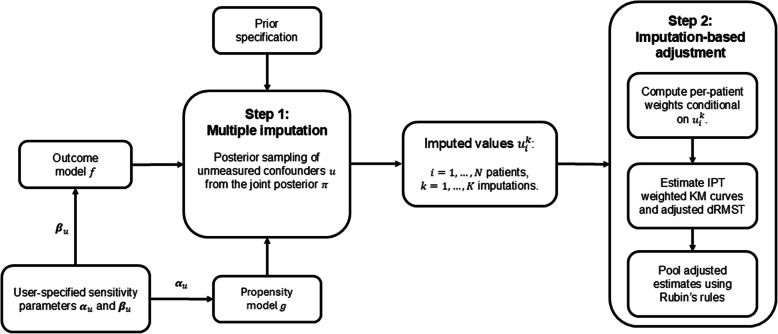
Proposed QBA framework with IPT weighting for adjustment to assess the sensitivity of dRMST to unmeasured confounding

Figure 1 summarises steps one and two of the proposed QBA framework. By iterating both steps for different pairs of association parameters $({\boldsymbol{\alpha }}_{u},{{\varvec{\beta}}}_{u}),$ the sensitivity of dRMST to unmeasured confounding can be assessed.

### Simulation study

#### Simulation model

The validity of our proposed imputation-based method for the adjustment of dRMST was assessed using a simulation study. Confounded data was simulated and analysed under a commonly encountered violation of the PH assumption, where a delayed treatment effect is observed in the intervention arm. In this scenario, survival is identical between arms until a specific time, after which treatment in the intervention arm becomes effective and survival is superior. While the proposed framework can account for the presence of both measured and unmeasured confounders, for simplicity we focus simulation analyses on an example where only unmeasured confounding is present.

Outcomes ${t}_{i}$ were simulated using a delayed treatment effect model with exponential survival. For patient $i=1,\dots ,N$ the hazard function was specified as:

7 $$h\left({t}_{i} \right|{z}_{i}, {u}_{i},{\beta }_{u},{\beta }_{z})=\lambda \text{exp(}{u}_{i}{\beta }_{u}\text{ + }{1({t}_{i}> {t}_{0})z}_{i}{\beta }_{z}\text{)},$$

where,

$$1\left({t}_{i}> {t}_{0}\right)=\left\{\begin{array}{c}0, {t}_{i} \le {t}_{0}, \\ 1, {t}_{i}> {t}_{0}.\end{array}\right.$$

In Eq. (7), ${u}_{i}$ denotes a binary confounder where ${u}_{i} \sim \text{Bernoulli(0.5)}$ and ${\beta }_{u}$ denotes its additive effect on the log hazard. The conditional HR capturing the effect of treatment was piecewise constant, and is given by:

8 $${\text{HR}}_{z}\text{ = }\frac{h\left(t \right| z=1, u,{\beta }_{u},{\beta }_{z})}{h\left(t \right| z=0, u,{\beta }_{u},{\beta }_{z})}\text{= exp(}{1}({t}_{i}> {t}_{0}){\beta }_{z}\text{)}.$$

Hence, survival between arms was identical (${\text{HR}}_{z}=1$) until a (known) change point ${t}_{0}$, after which treatment in the intervention arm becomes effective (${\text{HR}}_{z}\text{= exp(}{\beta }_{z}\text{) < 1}$)*.* As ${u}_{i}$ is a confounder we specified its association with ${z}_{i}$ using a logit link function defined by:

9 $$\text{Pr}\left({z}_{i} =1|{u}_{i}\right)={\text{logit}}^{-1}\left({\alpha }_{0}+{\alpha }_{1}{u}_{i}\right),{\boldsymbol{\alpha }}_{u}=\left({\alpha }_{0}, {\alpha }_{1}\right).$$

For the censoring process we applied administrative censoring at the end of the study period, denoted by $T$.

#### Simulation scenarios

Different simulation scenarios were considered by varying ${\beta }_{u}$, ${\alpha }_{0},$ and ${\alpha }_{1}.$ The values we considered were as follows: For ${\beta }_{u}$ we specified ${\beta }_{u}= \text{log(0.5)}$ or ${\beta }_{u}= \text{log(2)}$ which corresponds to a halving or doubling of the hazard respectively. Imbalance in $u$ was simulated by specifying four scenarios for (${\alpha }_{0},{\alpha }_{1}$). Two of these scenarios (small 1 and small 2) assigned a small imbalance between arms, and the remaining two (large 1 and large 2) assigned a large imbalance between arms. The values for (${\alpha }_{0},{\alpha }_{1}$) alongside the corresponding conditional probabilities of receiving treatment are displayed in Table 1.

**Table 1:** Conditional probabilities of receiving treatment under each imbalance scenario

Imbalance	(*α*_0_, *α*_1_)	Pr(*Z* = 1|*U* = 1)	Pr(*Z* = 1|*U* = 0)
Small 1	$(\text{log(}2/3\text{), }2\text{log(3/2))}$	0.6	0.4
Small 2	$(\text{log(3}/2\text{), }2\text{log(2/3))}$	0.4	0.6
Large 1	$(\text{log(1}/4\text{), }2\text{log(4))}$	0.8	0.2
Large 2	$(\text{log(4), }2\text{log(1/4))}$	0.2	0.8

Considering all possible combinations for ${\beta }_{u}$ and (${\alpha }_{0},{\alpha }_{1}$) gave a total of 8 simulation scenarios. In each scenario we set $N=300,$ $\lambda =0.073, { \beta }_{z}=\text{log(0.68)},$ ${t}_{0}= 5$, and $T = 48$. Under each scenario the dRMST adjusted for $u$ is readily computed (see supplementary material for further details). Irrespective of the imbalance in $u$ between arms the adjusted dRMST equals 3.59 when ${\beta }_{u}= \text{log(0.5)}$ and 2.36 when ${\beta }_{u}= \text{log(2).}$

#### Comparing adjustment methods

To optimally assess the characteristics of the proposed framework, we implemented the data generating model into the QBA framework, ensuring that model misspecification did not compromise evaluation of the framework’s validity. We compared dRMST estimates up to time horizon $\tau = 48$, obtained through adjustment using imputed values of $u$ against estimates adjusted using the actual simulated values of $u$. When adjusting using the actual $u$, dRMST was estimated through numerical integration of IPT weighted KM curves. To illustrate the magnitude of the bias induced by unmeasured confounding, a naive analysis was performed where the presence of confounding was ignored and the dRMST was estimated using numerical integration of unweighted KM curves. For each simulation scenario 100 datasets were simulated, with bias, standard error (SE) and 95% CI coverage compared between all three methods (Imputed, Actual, and Naive). Bias was defined as the difference between the estimated dRMST and the true adjusted dRMST. Implementation of imputation-based adjustment requires priors to be placed on all model parameters, to specify the joint posterior from which imputations are drawn. Therefore, the following priors were placed on all model parameters:

$$\text{log(}{\lambda}{\text)\sim N(}{\mu }_{0}\text{, }{\sigma }_{0}^{2}\text{),}$$

$${\beta }_{z} \, \sim \text{ N}\left({\mu }_{0}\text{, }{\sigma }_{0}^{2}\right),$$

$${u}_{i} \sim \text{Bernoulli(}\varphi \text{)},$$

$$\varphi \sim \text{Beta}(a_{\varphi },{b}_{\varphi }\text{),}$$

with ${\mu }_{0}$, ${\sigma }_{0}^{2}$, ${a}_{\varphi }$, and ${b}_{\varphi }$ set to the values given below to reflect vague prior beliefs:

$${\mu }_{0}=0,{ \sigma }_{0}^{2}=100,$$

$${a}_{\varphi }=1, { b}_{\varphi }=1.$$

MCMC sampling of the joint posterior was implemented using the statistical software JAGS and the R package rjags [59, 60] (see supplementary material for further details). For each simulated dataset a total of 5,000 posterior samples for ${u}_{i}$ were drawn. MCMC sampling efficiency was assessed by monitoring the effective sample size (ESS) [57], and through visual inspection of traceplots.

### Empirical application

Our proposed QBA framework was applied to data from a published immuno-oncology study which demonstrated clear violation of the PH assumption, through a delayed treatment effect for a novel immunotherapy. We used the proposed QBA framework to examine the sensitivity of the conclusions of this study to the presence of unmeasured confounding.

#### Description of dataset

The empirical dataset was taken from an ECA study which compared the efficacy of the CAR-T therapy brexucabtagene autoleucel, against standard-of-care therapies in patients with acute lymphoblastic leukaemia (ALL) [61, 62]. For the intervention arm, patients received brexucabtagene autoleucel as part of the single-arm ZUMA- 3 trial. To provide a comparator, an ECA (SCHOLAR- 3) was constructed from historical clinical trial data by the study authors, with propensity score matching used to identify suitable patients [63]. We focused on assessment of the overall survival (OS) results obtained in the ZUMA/SCHOLAR- 3 study. Specifically, we utilised data for the SCA- 2 sub-group, which included patients treated with the immunotherapies blinatumomab or inotuzumab from the SCHOLAR- 3 (*N*= 20) and ZUMA- 3 (*N*= 29) datasets as this subgroup showed clear PH-violation for OS [62]. As the ZUMA/SCHOLAR- 3 study was a non-randomised ECA analysis, unmeasured confounding may have been present if the matching process used by the original authors did not incorporate unmeasured covariates, which had an association with both treatment assignment and the study outcome.

#### Analysis of empirical data

As dRMST was not the effect measure of interest in the original ZUMA/SCHOLAR- 3 study, it was necessary to obtain such estimates before assessing their robustness to unmeasured confounding with QBA. Survival data was reconstructed using digitised data extracted from published KM curves [64]. Initial analyses were performed to estimate a reference dRMST, assuming the absence of unmeasured confounding through an unadjusted comparison of outcomes. Relative efficacy was summarised using numerical integration of KM curves to estimate the dRMST, and a 95% CI was computed using the method proposed by Conner et al. [46].

To assess the impact of unmeasured confounding our proposed QBA framework was applied to outcome data from the ZUMA/SCHOLAR- 3 study. For this analysis the outcome and propensity models were identical to those defined in the simulation study, with the exception that we assumed the presence of an unmeasured binary confounder $u \in \{-1, 1\}$ with user-specified associations ${\beta }_{u}$ and ${\alpha }_{u}$. A −1/1 coding for $u$ was considered as this removes the intercept from the propensity model, reducing the number of association parameters and simplifying visualisation of the output of the analysis. Therefore, the relationship between ${u}_{i}$ and treatment assignment is given by:

10 $${\text{Pr}}({z}_{i} = 1|{u}_{i}) = {\text{logit}}^{-1}(-{\alpha }_{u}{u}_{i}), {u}_{i}=-1, 1.$$

Under the above specification the corresponding odds ratio (OR) equals $\text{exp(}{2}{\alpha }_{u}\text{)}$. The conditional HR which captures the effect of $u$ on survival is given by:

11 $${\text{HR}}_{u}= \frac{{h}_{j}({t}_{i}|{u}_{i}=1, {z}_{i}=j)}{{h}_{j}({t}_{i}|{u}_{i}=-1, {z}_{i}=j)}= \frac{\text{exp(}{\beta }_{u}\text{)}}{\text{exp(}{-\beta }_{u}\text{)}}= \text{exp(2}{\beta }_{u}\text{)}, j=0, 1.$$

For the unknown parameters $\text{log(}\lambda )$, ${\beta }_{z}$, ${u}_{i}$ and $\varphi =\text{Pr}({u}_{i}=1)$, the prior specification remained the same as that used for the simulation study. As ${\alpha }_{u}$ and ${\beta }_{u}$ are user-specified, unique grid pair combinations (${\alpha }_{u}, {\beta }_{u}$) were specified, with the range of paired values chosen to reflect the extent of plausible associations that would be possessed by a binary confounder in practice. Values for ${\alpha }_{u}$ were set to $\left[-0.7,-0.6,\dots , 0.6, \left.0.7\right]\right.$ with the corresponding OR ranging between 0.25 to 4.06. Values for ${\beta }_{u}$ were set to $\left[-1,-0.9,\dots , 0.9, \left.1\right]\right.$ with the corresponding ${\text{HR}}_{u}$ ranging between 0.37 to 2.71. Using these values a grid of size $21\times 15=315$ was constructed, and the proposed framework was applied at each combination of grid points. For each adjusted analysis utilising a specific grid pair 5,000 posterior samples of $u$ were drawn, with a burn-in of 4,000 posterior samples leaving 1,000 imputed datasets.

For a fixed ${\alpha }_{u}$ the posterior estimates for ${\beta }_{z}, \lambda ,$ and $\varphi$ are expected to be similar for neighbouring grid point values of ${\beta }_{u}$. Therefore, to promote MCMC convergence, the sampled chains for ${\beta }_{z}, \lambda ,$ and $\varphi$ were initialised using posterior means estimated using MCMC output from the previous neighbouring grid point for ${\beta }_{u}$. At each grid point MCMC sampling efficiency was assessed by monitoring the ESS.

The objective of the analysis was to identify pairs (${\beta }_{u}, {\alpha }_{u}$) which induced a non-significant estimate (i.e. the 95% CI covers 0) for the adjusted dRMST. Pairs triggering a non-significant estimate were visualised using caterpillar plots, where the CIs for a given ${\alpha }_{u}$ are plotted across the range of values considered for ${\beta }_{u}$.

## Results

### Simulation study

**Fig. 2:**
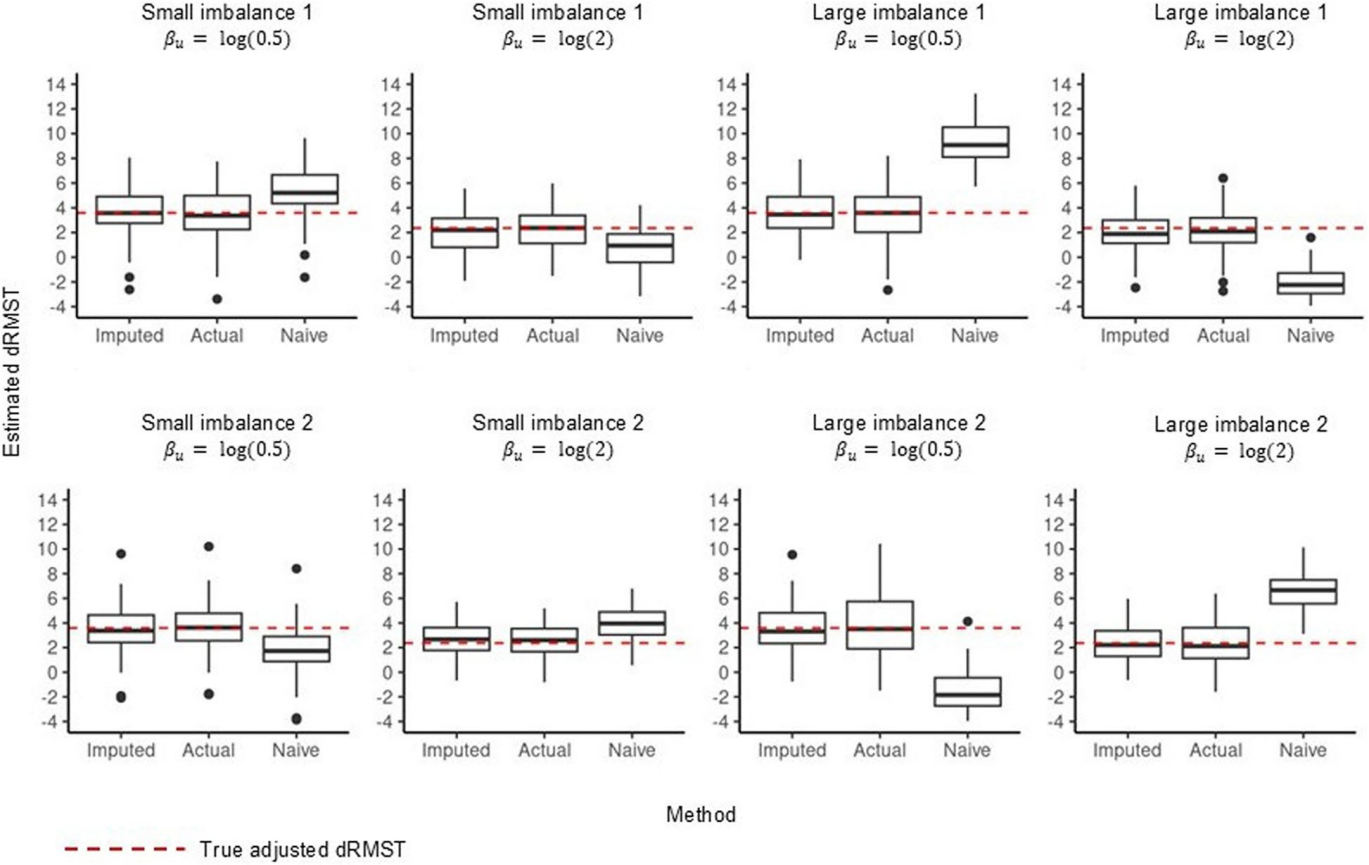
Output from the simulation study comparing the estimated dRMST between all 3 methods

The average ESS for $u$ under each simulation scenario was $>$ 100 indicating adequate mixing of the sampled chain for $u$, with visual inspection of traceplots suggesting that sampling occurred from the joint posterior across individual analyses (see supplementary material for further details). Figure 2 compares the estimated dRMST between all 3 methods. In Fig. 2 the naive analysis produced biased estimates across all 8 scenarios, with the magnitude of the bias being greater under large imbalances. In contrast, imputation-based adjustment was able to recover the true adjusted dRMST with minimal bias across all scenarios. Moreover, the ability to recover the true adjusted dRMST was comparable between imputation-based adjustment and actual adjustment across all scenarios. Imputation-based estimates were less precise when compared to actual adjustment, with a larger SE across all simulation scenarios (Table 2), reflecting the uncertainty in imputed values which is propagated into the pooled estimate. Examining the CI coverage rate in Table 2, the empirical rate exceeded the nominal rate of 95% when using imputation-based adjustment under large imbalances.

**Table 2:** Comparison of bias, SE, and CI coverage rate between methods imputed and actual

Simulation scenario		**Bias**^**a, b**^		**SE**^**a**^		**CI coverage rate**^**c**^	
${\boldsymbol\alpha}_u$ ^d^	${\beta }_{u}$ ^e^	Imputed	Actual	Imputed	Actual	Imputed	Actual
Small 1	$\text{log(0.5)}$	0.121	− 0.207	1.957	1.914	0.93	0.91
	$\text{log(2)}$	− 0.330	− 0.110	1.423	1.399	0.95	0.95
Small 2	$\text{log(0.5)}$	− 0.140	0.065	1.949	1.905	0.97	0.94
	$\text{log(2)}$	0.299	0.133	1.399	1.366	0.98	0.97
Large 1	$\text{log(0.5)}$	0.012	− 0.065	2.667	2.328	1.00	0.97
	$\text{log(2)}$	− 0.283	− 0.268	1.911	1.701	0.98	0.94
Large 2	$\text{log(0.5)}$	− 0.040	0.122	2.671	2.337	0.99	0.95
	$\text{log(2)}$	− 0.022	− 0.056	1.863	1.673	0.99	0.95

^a^Averaged over 100 simulations

^b^Bias as defined as estimate – truth

^c^Significance level = 95%

^d^Parameters for the logistic propensity model: Values induce the following imbalances:

Small 1: $$\text{Pr}(Z=1|U=1)=0.6$$, Small 2:$$\text{Pr}(Z=1|U=1)\boldsymbol{ }=\boldsymbol{ }0.4,$$

Large 1: $$\text{Pr}(Z=1|{U=1)}=\boldsymbol{ }0.8$$, Large 2:$$\text{Pr}(Z=\boldsymbol{ }1|U=1)\boldsymbol{ }=\boldsymbol{ }0.2.$$

^e^Conditional log(HR) capturing the effect of $u$ on survival: Values correspond to either a halving ($\text{log(0.5)}$)

or doubling ($\text{log(2)}$) of the hazard

### Empirical study

#### Estimation of dRMST assuming no unmeasured confounding

**Fig. 3:**
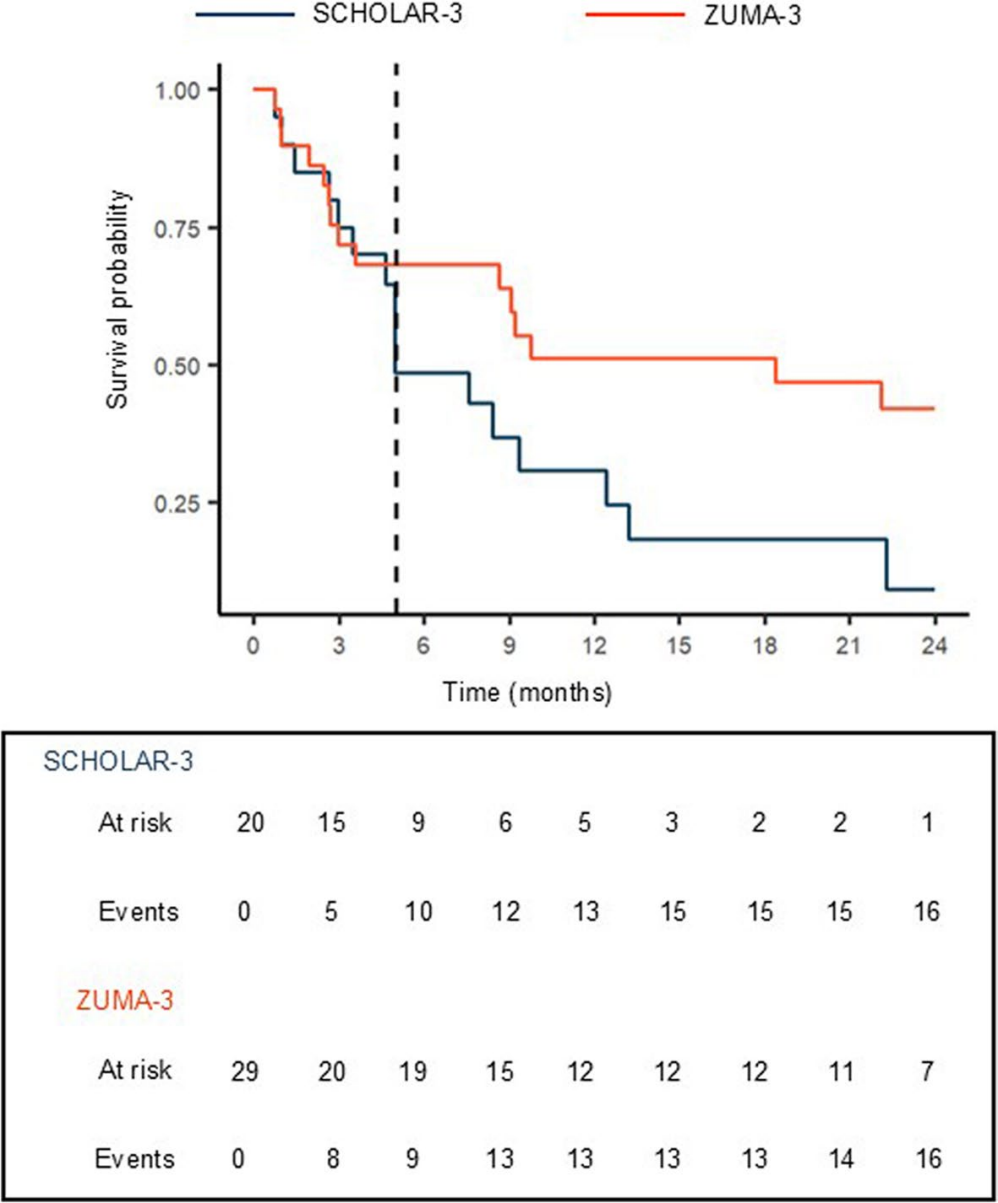
Estimated Kaplan-Meier curves for the ZUMA/SCHOLAR- 3 study

Figure 3 displays KM curves for the ZUMA/SCHOLAR- 3 study estimated using reconstructed survival data. Naive analysis of the ZUMA/SCHOLAR- 3 study resulted in a dRMST of 5.2 months; 95% CI: 0.07 - 10.2.

#### Application of proposed QBA framework

Implementation of the proposed QBA framework requires the specification of ${t}_{0}$ in the outcome model, which represents the time at which a treatment effect in the intervention arm is initiated. Visual assessment of Fig. 3 suggested that this occurred at a time of 5 months for the ZUMA/SCHOLAR- 3 study – denoted by the dashed vertical line in Fig. 3. Therefore, ${t}_{0}=5$ was specified in the outcome model for the analysis. The ESS averaged over all grid points for $\text{log(}\lambda )$ and ${\beta }_{z}$ was $\ge$ 100, which indicated acceptable MCMC mixing. The average ESS for $\varphi$ was $<$ 100 indicating relatively inefficient sampling of this parameter when compared to $\text{log}\left(\lambda \right)$ and ${\beta }_{z}$.

**Fig. 4:**
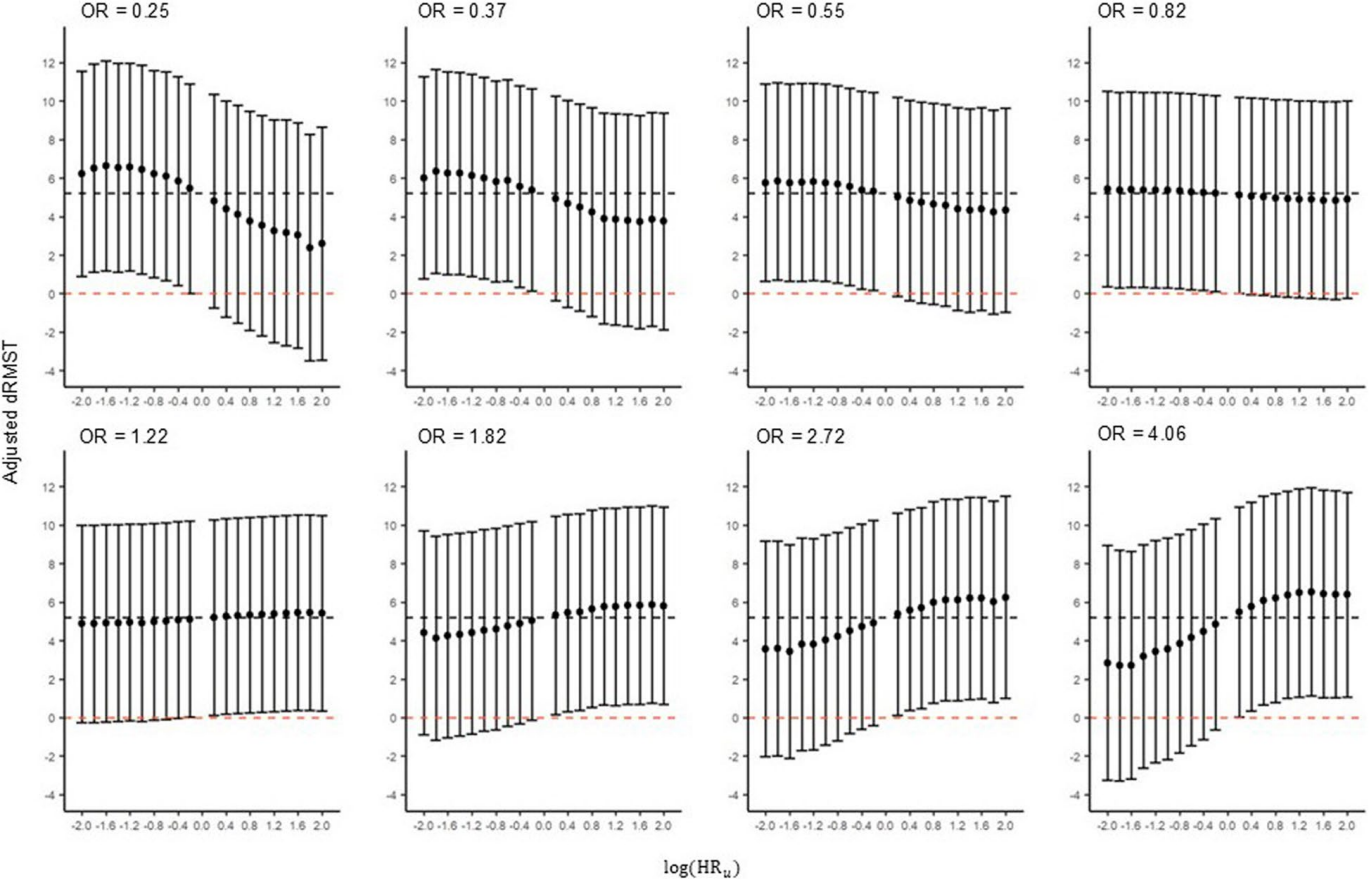
Output from our proposed QBA framework applied to the ZUMA/SCHOLAR- 3 study

Figure 4 presents adjusted dRMST results obtained by applying our proposed QBA framework to the ZUMA/SCHOLAR- 3 study across multiple pairs of values for the sensitivity parameters. As expected, when the effect of $u$ is small, i.e. OR $\approx$ 1 or ${\beta }_{u}\approx$ 0, an adjusted dRMST estimate is obtained which is close to the reference estimate (the estimate obtained assuming no unmeasured confounding – denoted by the dashed black line in Fig. 4). A non-significant dRMST estimate was obtained for the ZUMA/SCHOLAR- 3 study (Fig. 4) when OR $\le$ 0.82 and ${\text{HR}}_{u}$ $\ge$ 1.50, or when OR $\ge$ 1.22 and ${\text{HR}}_{u}\le$ 0.67, as seen at multiple points in Fig. 4 where the 95% CI crosses a dRMST of 0 – denoted by a dashed orange line.

## Discussion

In this article we have demonstrated a simulation-based QBA framework for TTE data that is valid in the presence of PH violation. The proposed framework uses Bayesian data augmentation to impute values for an unmeasured confounder with user-specified exposure and outcome associations, which are then used to re-weight survival data and compute confounder-adjusted dRMST estimates. Results from a simulation study demonstrated that the proposed adjustment method performs comparably when either an imputed or measured confounder is used, correctly identifying the confounder characteristics required to overturn a study’s conclusions.

The proposed framework was applied to the ZUMA/SCHOLAR- 3 ECA-based ITC study, which exhibited clear PH-violation. Visualising adjusted CIs across various exposure and outcome associations revealed confounding scenarios that nullified the ECA analysis conclusions, with the small sample size resulting in substantial uncertainty, as indicated by the wide adjusted dRMST CIs. Our results indicated that nullification would require an unmeasured confounder to decrease mortality by at least 33% with an OR for exposure above 1.22, or increase mortality by at least 50% with an OR below 0.82.

A key aspect of QBA is assessment of the plausibility of associations identified as being sufficient to nullify the study result. A common approach to achieve this is to benchmark the exposure and outcome associations against those estimated for a measured confounder. If the identified associations are larger than those observed for known confounders, it may be reasonable to conclude that unmeasured confounding is not strong enough to overturn the study results. For the ZUMA/SCHOLAR- 3 study, we did not have access to IPD, so it was not possible to estimate associations between known confounders and the exposure and outcome to conclusively assess the plausibility of the scenarios for which non-significant dRMST estimates were obtained. However, a form of contextualisation may be achieved by considering known prognostic markers of differential outcomes in ALL, that may act as confounders if unevenly distributed across the ECA and trial sources. For example, the Philadelphia chromosomal abnormality, present in ~ 20% of ALL cases, is associated with worse 5-year survival than other abnormalities (12% vs. 67%) [65]. These survival rates suggest that the greater than 50% hazard increase needed to nullify the study conclusions is plausible, but this would also simultaneously require a considerable imbalance in the distribution of this confounder between the ECA and trial sources (OR $\le$ 0.82). Moreover, given its strong prognostic significance, it is unlikely that such a confounder would go unidentified or unmeasured.

The QBA framework presented here was developed by extending previously proposed methods for simulation-based QBA [30], particularly for TTE data [29]. This extension involved replacing the expectation-maximisation algorithm previously used to impute unmeasured confounders with a Bayesian data augmentation approach. Despite utilising Bayesian data augmentation, the framework itself is not fully Bayesian, as the adjusted dRMST is estimated using frequentist methods. In this respect our framework is similar in structure to the two-step method proposed by Kaplan and Chen in [66], for uncertainty propagation in propensity score analyses. Fully Bayesian QBA frameworks have been previously proposed [32, 33] which enable specification of prior information for association parameters, in addition to generation of a summary of the adjusted effect estimate over all plausible association parameters in a marginal posterior estimate. Despite this, only relatively simple adjusted parametric survival distributions can be feasibly considered to compute a posterior estimate of the adjusted dRMST, therefore limiting the modelling flexibility of a fully Bayesian approach. Furthermore, as sources of unmeasured confounding may be unknown, the construction of informative priors for the association parameters may be a complex task, and if not correctly performed may result in estimation issues such as non-identifiability [31–33]. Our proposed QBA framework addresses these issues by employing a two-step estimation process that considers only user-specified sensitivity parameters, and separates the imputation process from treatment effect estimation. Separating the framework into these two distinct steps enables the use of flexible non-parametric methods for estimating the adjusted dRMST.

While alternative QBA methods that utilise dRMST have previously been introduced [47, 48], our proposed QBA framework is, to the best of our knowledge, the first simulation-based QBA method for the non-PH setting. In contrast to other QBA methods for dRMST, the Bayesian data augmentation framework presented here explicitly models confounding assumptions, rendering them transparent and easier to assess. By directly modelling confounder associations, the output becomes readily interpretable, with the identified associations reported using familiar effect measures (e.g., HR, OR). Moreover, the framework’s modular design permits implementation under user-specified outcome and propensity models, enabling a wide range of confounding assumptions to be considered. This flexibility extends to the choice of adjustment method, effect measure, and outcome model.

An additional advantage is that Bayesian data augmentation allows for the inclusion of prior information, enabling the integration of informative priors to address estimation issues associated with small sample sizes. Prior specification also facilitates the incorporation of external knowledge regarding the population distribution of the unmeasured confounder and its effect on survival. Finally, by generating a marginal posterior estimate of the population distribution of the unmeasured confounder, our framework provides further insight into the characteristics of a hypothetical unmeasured confounder, which may assist in evaluating the plausibility of unmeasured confounding.

While the proposed framework benefits from flexibility, it does possess some limitations. For example, extending the framework to incorporate more complex outcome models during the imputation step could result in posterior distributions that are challenging to sample using standard MCMC methods [67], potentially leading to inaccurate imputations and invalid results derived from poorly sampled unmeasured confounder imputations. Similarly, inaccurate imputation can result if the outcome or propensity model is misspecified. When the underlying relationships between the unmeasured confounder and either the outcome or treatment assignment are uncertain, we recommend implementing multiple sensitivity analyses using different forms of the unmeasured confounder and its relationships with the exposure and outcome to investigate the impact of these assumptions. Invalid results may also occur if the performance and characteristics of the selected adjustment method are not adequately assessed. For example, when using re-weighting methods, it is advisable to examine the presence of extreme treatment weights across all values in the grid search, as these can increase the variability of bias in effect estimates [17]. If extreme weights are identified, commonly applied methods, such as stabilisation and trimming [17, 68], can be incorporated into the framework. Similar considerations apply when using matching-based approaches for adjustment [69].

Use of the specific variance estimator for IPT weighted dRMST employed in our framework may also require careful consideration in practice. In the presence of strongly asymmetric exposure distributions, this estimator can lead to deviations from the nominal 95% confidence interval coverage [46]. Consequently, any QBA results obtained from the framework under such conditions should be interpreted with caution.

Finally, when applying the proposed framework, careful consideration must be given to what constitutes plausible magnitudes for confounder–exposure and confounder–outcome associations, as well as to determining an appropriate range of values for the grid search. Users should therefore ensure that the assumptions underlying each component of the framework are thoroughly evaluated, exercising caution when interpreting results in the presence of strongly asymmetric exposure distributions obtained using IPT weighting.

Despite being associated with considerable uncertainty due to the potential for unmeasured confounding, ITC evidence is increasingly being used in regulatory and reimbursement decision-making [3]. In recognition of these uncertainties, many regulatory bodies have issued guidance on best practices for analysing real world data and performing ITC analyses [21, 70, 71]. A unifying theme of these guidelines is the critical role played by sensitivity analysis and QBA in contextualising the inherent uncertainty involved when making decisions based on this type of evidence. Therefore, it is vital that suitable QBA frameworks are available for any analysis scenario that may be encountered when generating ITC evidence. Although many QBA methods have been proposed to assess the robustness of a study to unmeasured confounding, these methods must be valid under PH violation to ensure their applicability across the breadth of scenarios that may arise in the regulatory assessment of a novel therapy. The framework presented here is valid under PH violation and can play a fundamental role when performing sensitivity analysis for ITCs, contributing to the reduction of uncertainty in the regulatory and reimbursement decision-making process for novel therapies.

## Supplementary Information

Supplementary Material 1.

Supplementary Material 2.

## Data Availability

No datasets were generated or analysed during the current study.

## Abbreviations

ALL: Acute Lymphoblastic Leukaemia
CI: Confidence Interval
dRMST: Difference in Restricted Mean Survival
ECA: External Control Arm
EHR: Electronic Health Records
ESS: Effective Sample Size
IPD: Individual Patient Data
IPT: Inverse Probability of Treatment
ITC: Indirect Treatment Comparison
HR: Hazard Ratio
KM: Kaplan-Meier
MCMC: Markov Chain Monte Carlo
MI: Multiple Imputation
OR: Odds Ratio
OS: Overall Survival
PH: Proportional Hazards
PSM: Propensity Score Matching
QBA: Quantitative Bias Analysis
RCT: Randomised Controlled Trial
RMST: Restricted Mean Survival
SE: Standard Error
TTE: Time-To-Event

## References

[1] Hariton E, Locascio JJ. Randomised controlled trials—the gold standard for effectiveness research. BJOG Int J Obstet Gynaecol. 2018;125:1716. 10.1111/1471-0528.15199 PMC6235704 29916205

[2] Wang M, Ma H, Shi Y, Ni H, Qin C, Ji C. Single-arm clinical trials: design, ethics, principles. BMJ Support Palliat Care. 2024. 10.1136/spcare-2024-004984. 38834238 10.1136/spcare-2024-004984 PMC11874317

[3] Patel D, Grimson F, Mihaylova E, Wagner P, Warren J, van Engen A, et al. Use of external comparators for health technology assessment submissions based on single-arm trials. Value Health. 2021;24:1118–25. 34372977 10.1016/j.jval.2021.01.015

[4] Davi R, Mahendraratnam N, Chatterjee A, Dawson CJ, Sherman R. Informing single-arm clinical trials with external controls. Nat Rev Drug Discov. 2020;19:821–2. 32811986 10.1038/d41573-020-00146-5

[5] Makady A, de Boer A, Hillege H, Klungel O, Goettsch W. What Is real-world data? A review of definitions based on literature and stakeholder interviews. Value Health. 2017;20:858–65. 28712614 10.1016/j.jval.2017.03.008

[6] Liu F, Panagiotakos D. Real-world data: a brief review of the methods, applications, challenges and opportunities. BMC Med Res Methodol. 2022;22:287. 36335315 10.1186/s12874-022-01768-6 PMC9636688

[7] Signorovitch JE, Wu EQ, Yu AP, Gerrits CM, Kantor E, Bao Y, et al. Comparative effectiveness without head-to-head trials. PharmacoEconomics. 2010;28:935–45. 20831302 10.2165/11538370-000000000-00000

[8] Phillippo DM, Ades AE, Dias S, Palmer S, Abrams KR, Welton NJ. Methods for population-adjusted indirect comparisons in health technology appraisal. Med Decis Making. 2018;38:200–11. 28823204 10.1177/0272989X17725740 PMC5774635

[9] Mishra-Kalyani PS, Kordestani LA, Rivera DR, Singh H, Ibrahim A, DeClaro RA, et al. External control arms in oncology: current use and future directions. Ann Oncol. 2022;33:376–83. 35026413 10.1016/j.annonc.2021.12.015

[10] Loiseau N, Trichelair P, He M, Andreux M, Zaslavskiy M, Wainrib G, et al. External control arm analysis: an evaluation of propensity score approaches, G-computation, and doubly debiased machine learning. BMC Med Res Methodol. 2022;22:335. 36577946 10.1186/s12874-022-01799-z PMC9795588

[11] Seeger JD, Davis KJ, Iannacone MR, Zhou W, Dreyer N, Winterstein AG, et al. Methods for external control groups for single arm trials or long-term uncontrolled extensions to randomized clinical trials. Pharmacoepidemiol Drug Saf. 2020;29:1382–92. 32964514 10.1002/pds.5141 PMC7756307

[12] Sutton A, Ades AE, Cooper N, Abrams K. Use of indirect and mixed treatment comparisons for technology assessment. PharmacoEconomics. 2008;26:753–67. 18767896 10.2165/00019053-200826090-00006

[13] Hernán MA, Robins JM. Using big data to emulate a target trial when a randomized trial is not available. Am J Epidemiol. 2016;183:758–64. 26994063 10.1093/aje/kwv254 PMC4832051

[14] Hernan M, Robins J. What if. Boca Raton: Chapman and Hill/CRC; 2020.

[15] Rosenbaum PR, Rubin DB. The central role of the propensity score in observational studies for causal effects. Biometrika. 1983;70:41–55.

[16] Bang H, Robins JM. Doubly robust estimation in missing data and causal inference models. Biometrics. 2005;61:962–73. 16401269 10.1111/j.1541-0420.2005.00377.x

[17] Cole SR, Hernán MA. Constructing inverse probability weights for marginal structural models. Am J Epidemiol. 2008;168:656–64. 18682488 10.1093/aje/kwn164 PMC2732954

[18] Naimi AI, Cole SR, Kennedy EH. An introduction to g methods. Int J Epidemiol. 2017;46:756–62. 28039382 10.1093/ije/dyw323 PMC6074945

[19] Sammon CJ, Leahy TP, Gsteiger S, Ramagopalan S. Real-world evidence and nonrandomized data in health technology assessment: using existing methods to address unmeasured confounding? J Comp Eff Res. 2020;9:969–72. 32757772 10.2217/cer-2020-0112

[20] Groenwold RHH, Hak E, Hoes AW. Quantitative assessment of unobserved confounding is mandatory in nonrandomized intervention studies. J Clin Epidemiol. 2009;62:22–8. 18619797 10.1016/j.jclinepi.2008.02.011

[21] NICE. NICE real-world evidence framework. 2022. https://www.nice.org.uk/corporate/ecd9/chapter/overview. Accessed 2 Feb 2024.

[22] Uddin MdJ, Groenwold RHH, Ali MS, de Boer A, Roes KCB, Chowdhury MAB, et al. Methods to control for unmeasured confounding in pharmacoepidemiology: an overview. Int J Clin Pharm. 2016;38:714–23. 27091131 10.1007/s11096-016-0299-0

[23] Arah OA. Bias analysis for uncontrolled confounding in the health sciences. Annu Rev Public Health. 2017;38:23–38. 28125388 10.1146/annurev-publhealth-032315-021644

[24] Zhang X, Faries DE, Li H, Stamey JD, Imbens GW. Addressing unmeasured confounding in comparative observational research. Pharmacoepidemiol Drug Saf. 2018;27:373–82. 29383840 10.1002/pds.4394

[25] D’Agostino McGowan L. Sensitivity analyses for unmeasured confounders. Curr Epidemiol Rep. 2022;9:361–75.

[26] VanderWeele TJ, Ding P. Sensitivity analysis in observational research: introducing the E-value. Ann Intern Med. 2017;167:268–74. 28693043 10.7326/M16-2607

[27] VanderWeele TJ, Arah OA. Bias formulas for sensitivity analysis of unmeasured confounding for general outcomes, treatments, and confounders. Epidemiology. 2011;22:42. 21052008 10.1097/EDE.0b013e3181f74493 PMC3073860

[28] Lin DY, Psaty BM, Kronmal RA. Assessing the sensitivity of regression results to unmeasured confounders in observational studies. Biometrics. 1998;54:948–63. 9750244

[29] Huang R, Xu R, Dulai PS. Sensitivity analysis of treatment effect to unmeasured confounding in observational studies with survival and competing risks outcomes. Stat Med. 2020;39:3397–411. 32677758 10.1002/sim.8672

[30] Carnegie NB, Harada M, Hill JL. Assessing sensitivity to unmeasured confounding using a simulated potential confounder. J Res Educ Eff. 2016;9:395–420.

[31] Dorie V, Harada M, Carnegie NB, Hill J. A flexible, interpretable framework for assessing sensitivity to unmeasured confounding. Stat Med. 2016;35:3453–70. 27139250 10.1002/sim.6973 PMC5084780

[32] McCandless LC, Gustafson P, Levy A. Bayesian sensitivity analysis for unmeasured confounding in observational studies. Stat Med. 2007;26:2331–47. 16998821 10.1002/sim.2711

[33] McCandless LC, Gustafson P, Levy AR, Richardson S. Hierarchical priors for bias parameters in Bayesian sensitivity analysis for unmeasured confounding. Stat Med. 2012;31:383–96. 22253142 10.1002/sim.4453

[34] Thorlund K, Duffield S, Popat S, Ramagopalan S, Gupta A, Hsu G, et al. Quantitative bias analysis for external control arms using real-world data in clinical trials: a primer for clinical researchers. J Comp Eff Res. 13(3):e230147. 10.57264/cer-2023-0147 PMC10945419 38205741

[35] Rahman R, Fell G, Ventz S, Arfé A, Vanderbeek AM, Trippa L, et al. Deviation from the proportional hazards assumption in randomized phase 3 clinical trials in oncology: prevalence, associated factors, and implications. Clin Cancer Res. 2019;25:6339–45. 31345838 10.1158/1078-0432.CCR-18-3999

[36] Rahman R, Fell G, Trippa L, Alexander BM. Violations of the proportional hazards assumption in randomized phase III oncology clinical trials. J Clin Oncol. 2018;36 15_suppl:2543–2543.

[37] Salmon D, Melendez-Torres GJ. Clinical effectiveness reporting of novel cancer drugs in the context of non-proportional hazards: a review of nice single technology appraisals. Int J Technol Assess Health Care. 2023;39:e16. 36883316 10.1017/S0266462323000119 PMC11574539

[38] Lin TA, McCaw ZR, Koong A, Lin C, Abi Jaoude J, Patel R, et al. Proportional hazards violations in phase III cancer clinical trials: a potential source of trial misinterpretation. Clin Cancer Res Off J Am Assoc Cancer Res. 2024;30:4791–9. 10.1158/1078-0432.CCR-24-0566 PMC11479825 39133081

[39] Chen T-T. Statistical issues and challenges in immuno-oncology. J Immunother Cancer. 2013;1:18. 24829754 10.1186/2051-1426-1-18 PMC4019889

[40] Hsu CY, Lin EPY, Shyr Y. Development and evaluation of a method to correct misinterpretation of clinical trial results with long-term survival. JAMA Oncol. 2021;7:1041–4. 33856410 10.1001/jamaoncol.2021.0289 PMC8050786

[41] Uno H, Claggett B, Tian L, Inoue E, Gallo P, Miyata T, et al. Moving beyond the hazard ratio in quantifying the between-group difference in survival analysis. J Clin Oncol. 2014;32:2380–5. 24982461 10.1200/JCO.2014.55.2208 PMC4105489

[42] Pak K, Uno H, Kim DH, Tian L, Kane RC, Takeuchi M, et al. Interpretability of cancer clinical trial results using restricted mean survival time as an alternative to the hazard ratio. JAMA Oncol. 2017;3:1692–6. 28975263 10.1001/jamaoncol.2017.2797 PMC5824272

[43] Royston P. Estimating the treatment effect in a clinical trial using difference in restricted mean survival time. Stata J. 2015;15:1098–117.

[44] Ambrogi F, Iacobelli S, Andersen PK. Analyzing differences between restricted mean survival time curves using pseudo-values. BMC Med Res Methodol. 2022;22:71. 35300614 10.1186/s12874-022-01559-z PMC8931966

[45] Cole SR, Hernán MA. Adjusted survival curves with inverse probability weights. Comput Methods Programs Biomed. 2004;75:45–9. 15158046 10.1016/j.cmpb.2003.10.004

[46] Conner SC, Sullivan LM, Benjamin EJ, LaValley MP, Galea S, Trinquart L. Adjusted restricted mean survival times in observational studies. Stat Med. 2019;38:3832–60. 31119770 10.1002/sim.8206 PMC7534830

[47] Lin Z, Ni A, Lu B. Matched design for marginal causal effect on restricted mean survival time in observational studies. J Causal Inference. 2023;11:20220035.

[48] Lee S, Park JH, Lee W. Sensitivity analysis for unmeasured confounding in estimating the difference in restricted mean survival time. Stat Methods Med Res. 2024;33:1979–92. 39371030 10.1177/09622802241280782

[49] Lin NX, Logan S, Henley WE. Bias and sensitivity analysis when estimating treatment effects from the Cox model with omitted covariates. Biometrics. 2013;69:850–60. 24224574 10.1111/biom.12096 PMC4230475

[50] VanderWeele TJ. Unmeasured confounding and hazard scales: sensitivity analysis for total, direct, and indirect effects. Eur J Epidemiol. 2013;28:113–7. 23371044 10.1007/s10654-013-9770-6 PMC3606287

[51] Klungsøyr O, Sexton J, Sandanger I, Nygård JF. Sensitivity analysis for unmeasured confounding in a marginal structural Cox proportional hazards model. Lifetime Data Anal. 2009;15:278–94. 19109770 10.1007/s10985-008-9109-x

[52] Ananthakrishnan R, Green S, Previtali A, Liu R, Li D, LaValley M. Critical review of oncology clinical trial design under non-proportional hazards. Crit Rev Oncol Hematol. 2021;162:103350. 33989767 10.1016/j.critrevonc.2021.103350

[53] Greenland S. Multiple-bias modelling for analysis of observational data. J R Stat Soc Ser A Stat Soc. 2005;168:267–306.

[54] van Dyk DA, Meng X-L. The art of data augmentation. J Comput Graph Stat. 2001;10:1–50.

[55] Dempster AP, Laird NM, Rubin DB. Maximum likelihood from incomplete data via the EM algorithm. J R Stat Soc Ser B Methodol. 1977;39:1–22.

[56] Tanner MA, Wong WH. The calculation of posterior distributions by data augmentation. J Am Stat Assoc. 1987;82:528–40.

[57] Brooks S, Gelman A, Jones G, Meng XL. Handbook of Markov Chain Monte Carlo. Boca Raton: CRC Press; 2011.

[58] Austin PC, White IR, Lee DS, van Buuren S. Missing data in clinical research: a tutorial on multiple imputation. Can J Cardiol. 2021;37:1322–31. 33276049 10.1016/j.cjca.2020.11.010 PMC8499698

[59] Plummer M, Stukalov A, Denwood M. rjags: Bayesian Graphical Models using MCMC. 2023.

[60] Plummer M. JAGS: a program for analysis of Bayesian graphical models using Gibbs sampling. 2023.

[61] Shah BD, Ghobadi A, Oluwole OO, Logan AC, Boissel N, Cassaday RD, et al. KTE-X19 for relapsed or refractory adult B-cell acute lymphoblastic leukaemia: phase 2 results of the single-arm, open-label, multicentre ZUMA-3 study. Lancet. 2021;398:491–502. 34097852 10.1016/S0140-6736(21)01222-8 PMC11613962

[62] Shah BD, Ghobadi A, Oluwole OO, Logan AC, Boissel N, Cassaday RD, et al. Two-year follow-up of KTE-X19 in patients with relapsed or refractory adult B-cell acute lymphoblastic leukemia in ZUMA-3 and its contextualization with SCHOLAR-3, an external historical control study. J Hematol Oncol. 2022;15:170. 36494725 10.1186/s13045-022-01379-0 PMC9734710

[63] Shah BD, Faghmous I, Whitmore J, Masouleh BK, Xu H. The comparison of Kte-X19 to current standards of care: a pre-specified synthetic control study utilizing individual patient level data from historic clinical trials (SCHOLAR-3). Blood. 2021;138:3844.

[64] Liu N, Zhou Y, Lee JJ. IPDfromKM: reconstruct individual patient data from published Kaplan-Meier survival curves. BMC Med Res Methodol. 2021;21:111. 34074267 10.1186/s12874-021-01308-8 PMC8168323

[65] Kantarjian H, Thomas D, O’Brien S, Cortes J, Giles F, Jeha S, et al. Long-term follow-up results of hyperfractionated cyclophosphamide, vincristine, doxorubicin, and dexamethasone (Hyper-CVAD), a dose-intensive regimen, in adult acute lymphocytic leukemia. Cancer. 2004;101:2788–801. 15481055 10.1002/cncr.20668

[66] Kaplan D, Chen J. A two-step Bayesian approach for propensity score analysis: simulations and case study. Psychometrika. 2012;77:581–609. 27519782 10.1007/s11336-012-9262-8

[67] Matsuura K. How to Improve MCMC ConvergenceMCMC convergence. In: Matsuura K, editor. Bayesian Statistical Modeling with Stan, R, and Python. Singapore: Springer Nature; 2022. p. 183–212.

[68] Austin PC, Stuart EA. Moving towards best practice when using inverse probability of treatment weighting (IPTW) using the propensity score to estimate causal treatment effects in observational studies. Stat Med. 2015;34:3661–79. 26238958 10.1002/sim.6607 PMC4626409

[69] Ishak KJ, Proskorovsky I, Benedict A. Simulation and matching-based approaches for indirect comparison of treatments. PharmacoEconomics. 2015;33:537–49. 25795232 10.1007/s40273-015-0271-1

[70] European Medicines Agency. Real-world evidence framework to support EU regulatory decision-making. 2023.

[71] Commissioner O of the. Real-World Evidence. FDA. 2023. https://www.fda.gov/science-research/science-and-research-special-topics/real-world-evidence. Accessed 4 Sep 2023.

