# Supplementary material for: Flexible quantitative bias analysis for unmeasured confounding in subject-level indirect treatment comparisons with proportional hazards violation
Source: BMC Med Res Methodol. 2025 May 10;25:131. doi: 10.1186/s12874-025-02551-z (PMC12066054; doi:10.1186/s12874-025-02551-z)
Supplement: Supplementary file 1 — Supplementary Material 1. [file 12874_2025_2551_MOESM1_ESM.docx]

**Supplementary Material**

**Adjustment of dRMST using IPT Weighting**

Let $j=1,\ldots,J$ index follow-up times were at least one event occurred. Adjusted KM curves are estimated for arm $z=0,1$ by first weighting the number of patients who experienced the event or were still at risk at time $t_{j}$:

$$y_{j, z}^{'}= \sum_{i:T_{i}\geq t_{j}} w_{i}\boldsymbol{1}(z_{i}=z),$$

$$d_{j, z}^{'}= \sum_{i:T_{i}=t_{j}} w_{i}\delta_{i}\boldsymbol{1}(z_{i}=z),$$

where $T_{i}$, $\delta_{i}$, and $w_{i}$ denote the outcome, event indicator and propensity score for patient $i$ respectively. The adjusted KM curve at time $t$ is then given by:

$\hat{S_{z}}\left( t \right)= \sum_{t_{j}\leq t} \left[ 1- \frac{d_{j, z}^{'}}{y_{j, z}^{'}} \right], z=0, 1.$ (S1)

An estimate of the adjusted RMST up to time horizon $\tau$ is then given by:

$$\hat{\mu_{z}}=\int_{0}^{\tau} \hat{S_{z}}\left( t \right)\text{d}t, z=0,1,$$

where the above integral is computed using numerical integration. The adjusted dRMST is then estimated as $\hat{\nu}=\hat{\mu_{1}}-\hat{\mu_{0}}$. A variance estimator for the adjusted RMST was proposed by Conner et al (1) and is given by:

$\hat{V}\left( \hat{\mu_{z}} \right)=\sum_{j:t_{j}\leq\tau} \left[ \sum_{i=j}^{\tau} \hat{S_{z}}(t_{i})(t_{i+1}-t_{i}) \right]^{2}\frac{d_{j, z}^{'}}{M_{j,z}(y_{j, z}^{'}-d_{j, z}^{'})}, z=0, 1,$ (S2)

where,

$$M_{j,z}=\frac{\left[ \sum_{i:T_{i}\geq t_{j}} w_{i}\boldsymbol{1}(z_{i}=z) \right]^{2}}{\sum_{i:T_{i}\geq t_{j}} w_{i}^{2}\boldsymbol{1}(z_{i}=z)}.$$

As the adjusted RMSTs are independent the variance of the adjusted dRMST is estimated by:

$\hat{V}\left( \hat{\nu} \right)= \hat{V}\left( \hat{\mu_{1}} \right)+ \hat{V}\left( \hat{\mu_{0}} \right).$ (S3)

**True Adjusted dRMST**

Computation of the true adjusted dRMST first requires calculation of conditional survival curves. Under the delayed treatment effect model with exponential survival, conditional survival curves are given by:

$S(t|z,\lambda,\beta_{z},\beta_{u}, u)= \text{exp}\left\{ \text{-}\text{H}(t|z,\lambda,\beta_{z}, \beta_{u}, u) \right\}, u=0,1,$ (S4)

where $H(t|z,\lambda,\beta_{z}, u)$ denotes the cumulative hazard function conditional on $u$ defined for each arm by:

$H(t|z = 0,\lambda,\beta_{z}, \beta_{u}, u)=\lambda t\text{exp(}u\beta_{u}),$ (S5)

$H(t|z = 1,\lambda,\beta_{z},\beta_{u}, u)=\lambda t\text{exp(}u\times\beta_{u}) + \lambda(t - t_{0})\text{exp(}u\beta_{u}+\beta_{z})\times\boldsymbol{1}(t - t_{0}), u=0, 1$, (S6)

where,

$$\boldsymbol{1}(t - t_{0})=\left\{ \begin{aligned} 0 0\leq t\leq t_{0}, \\ 1 t >t_{0}. \end{aligned} \right.$$

Adjusted survival curves for each arm are then obtained by marginalisation of $u$ as follows:

$S(t|\lambda,\beta_{z},\beta_{u})=\sum_{u=0}^{1} S(t|z,\lambda,\beta_{z},\beta_{u}, u), z=0,1.$ (S7)

Adjusted survival is computed for a range of points in $t \in\left[ 0,\left. \tau\right] \right.$ and numerical integration then used to compute the adjusted RMST up to $\tau$ for each arm. The adjusted dRMST is then the difference between adjusted RMSTs.

**MCMC Diagnostics**

Table A1 displays the effective sample size (ESS) for selected parameters in the simulation study.

| Table A1: ESS^1^ for selected parameters under each simulation scenario. | | | | |
| --- | --- | --- | --- | --- |
| Simulation scenario | |  | Parameter |  |
| $\boldsymbol{\alpha}_{\boldsymbol{u}}^{\boldsymbol{2}}$ | $\beta_{u}^{3}$ | $\lambda$ | $\beta_{z}$ | $u_{i}^{4}$ |
| Small 1 | $log(0.5)$ | 43.30 | 496.62 | 445.99 |
|  | $log(2)$ | 44.88 | 519.17 | 471.72 |
| Small 2 | $log(0.5)$ | 50.90 | 522.46 | 445.32 |
|  | $log(2)$ | 43.64 | 461.87 | 461.83 |
| Large 1 | $log(0.5)$ | 423.03 | 559.39 | 987.18 |
|  | $log(2)$ | 463.41 | 507.38 | 984.39 |
| Large 2 | $log(0.5)$ | 466.34 | 547.24 | 986.23 |
|  | $log(2)$ | 386.66 | 558.24 | 985.09 |
| 1: Averaged over 100 simulations.  2: Parameters for the logistic propensity model: Values induce the following imbalances: Small 1: $\mathbf{Pr}\boldsymbol{(Z = 1\vert U = 1) =0.4}$, Small 2: $\mathbf{Pr(}\boldsymbol{Z = 1\vert U=1) = 0.6,}$  Large 1: $\mathbf{Pr}\boldsymbol{(Z = 1\vert U=1) = 0.2}$, Large 2: $\mathbf{Pr}\boldsymbol{(Z = 1\vert U=1) = 0.8.}$ 3: Conditional log(HR) capturing the effect of $\boldsymbol{u}$ on survival: Values correspond to either a halving ($\text{log(0.5)}$)  or doubling ($\text{log(2)}$) of the hazard. 4: Averaged over all patients and simulations. | | | | |

Figures A1 and A2 display thinned traceplots of the sampled chains for the parameters $\text{log(λ)}$ and $\beta_{z}$ respectively.

**Figure A1**: Thinned traceplots of the sampled chains for $\text{log(λ)}$ across simulation scenarios.


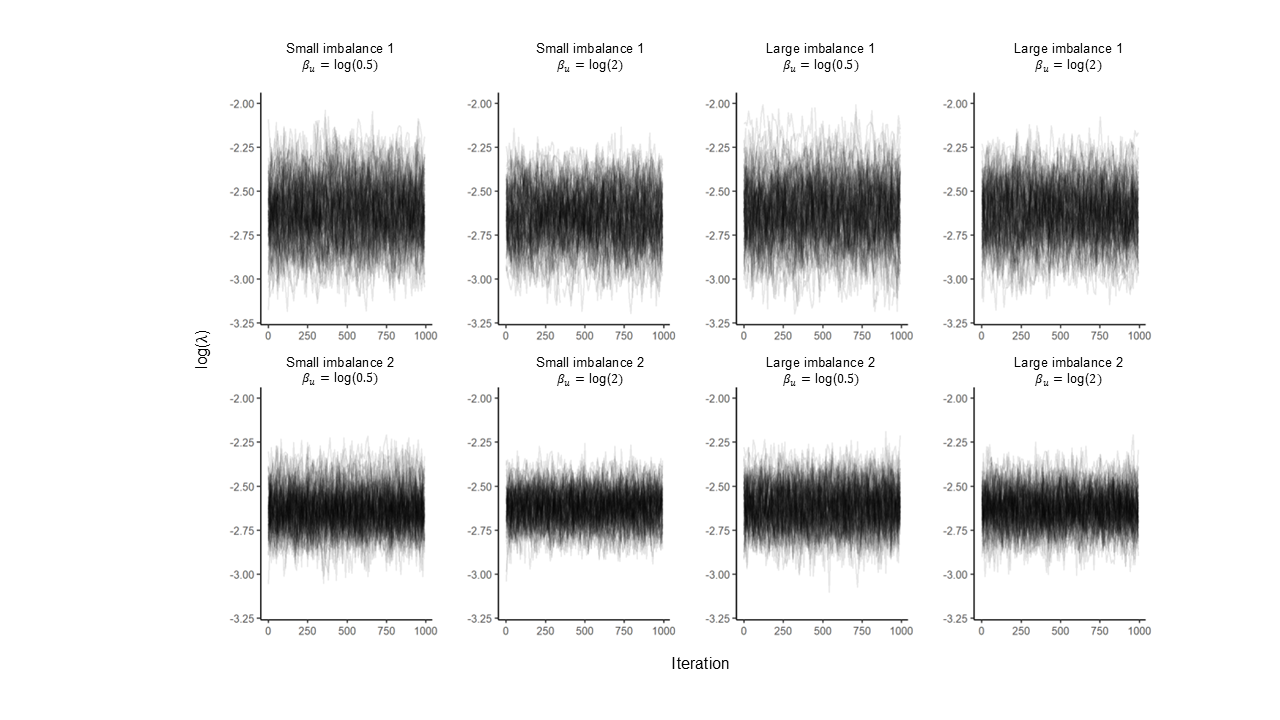


**Figure A2**: Thinned traceplots of the sampled chain for $\beta_{z}$ across simulation scenarios.


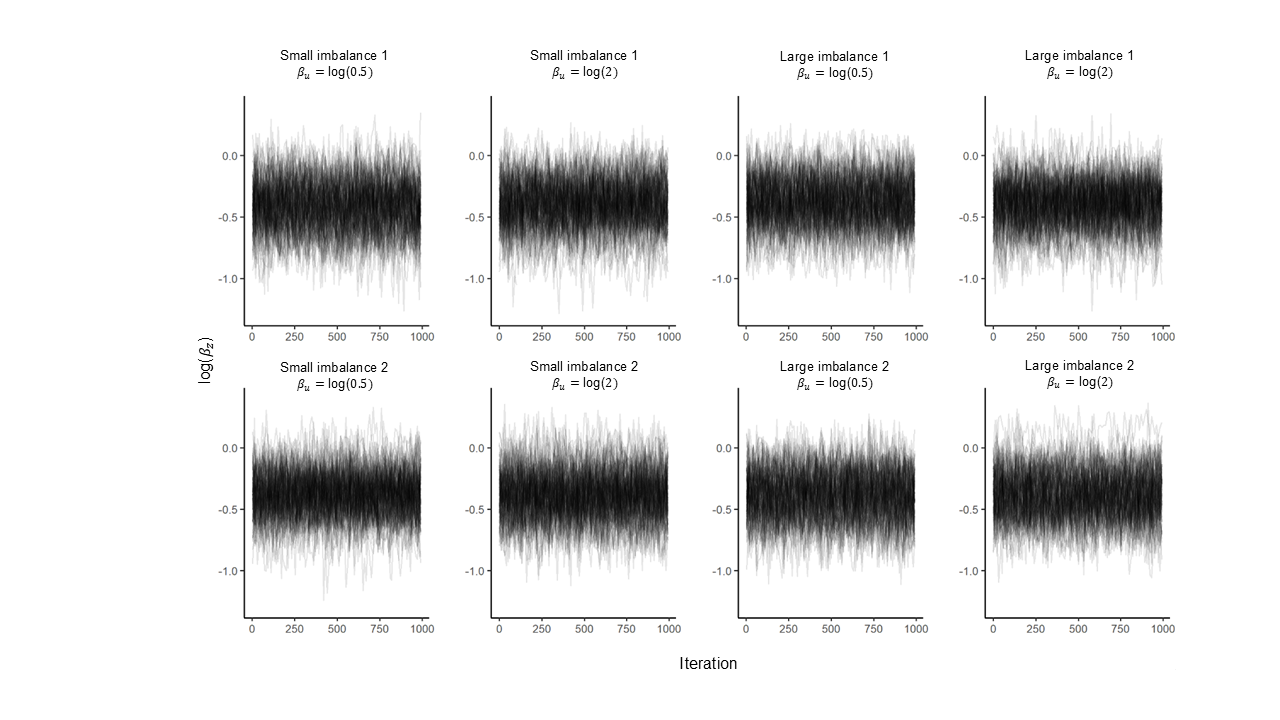


**References**

1. Conner SC, Sullivan LM, Benjamin EJ, LaValley MP, Galea S, Trinquart L. Adjusted restricted mean survival times in observational studies. Statistics in Medicine. 2019;38(20):3832–60.
